# Recombinant Antibodies with Unique Specificities Allow for Sensitive and Specific Detection of Uncarboxylated Osteocalcin in Human Circulation

**DOI:** 10.1007/s00223-020-00746-8

**Published:** 2020-08-24

**Authors:** Milja Arponen, Eeva-Christine Brockmann, Riku Kiviranta, Urpo Lamminmäki, Kaisa K. Ivaska

**Affiliations:** 1 Institute of Biomedicine, University of Turku, 20520 Turku, Finland; 2 Department of Biotechnology, University of Turku, Turku, Finland

**Keywords:** Bone, Osteocalcin, Uncarboxylated osteocalcin, Glucose, Type 2 diabetes

## Abstract

Osteocalcin is a bone-specific protein which contains three glutamic acid residues (Glu) that undergo post-translational gamma-carboxylation. Uncarboxylated osteocalcin (ucOC) may participate in the regulation of glucose metabolism, thus measurement of ucOC could be useful in evaluating interactions between bone and glucose metabolism. We developed recombinant antibodies and immunoassay to specifically detect ucOC in human blood samples. ucOC-specific recombinant antibodies were selected from an antibody library by phage display. Four candidates were characterized, and one (Fab-AP13) was used to set up an immunoassay with a pre-existing MAb. Plasma ucOC levels were measured in subjects with normal fasting blood glucose (≤ 6 mmol/l, *N* = 46) or with hyperglycemia (≥ 7 mmol/l, *N* = 29). Further, we analyzed ucOC in age- and gender-matched patients with diagnosed type 2 diabetes (T2D, *N* = 49). Antibodies recognized ucOC without cross-reaction to carboxylated osteocalcin. Antibodies had unique binding sites at the carboxylation region, with Glu17 included in all epitopes. Immunoassay was set up and characterized. Immunoassay detected ucOC in serum and plasma, with on average 1.6-fold higher levels in plasma. ucOC concentrations were significantly lower in subjects with hyperglycemia (median 0.58 ng/ml, *p* = 0.008) or with T2D diagnosis (0.68 ng/ml, *p* = 0.015) than in subjects with normal blood glucose (1.01 ng/ml). ucOC negatively correlated with fasting plasma glucose in subjects without T2D (*r* = − 0.24, *p* = 0.035) but not in T2D patients (*p* = 0.41). Our immunoassay, based on the novel recombinant antibody, allows for specific and sensitive detection of ucOC in human circulation. Correlation between ucOC and plasma glucose suggests interactions between osteocalcin and glucose metabolism in humans.

**Electronic supplementary material:**

The online version of this article (10.1007/s00223-020-00746-8) contains supplementary material, which is available to authorized users.

## Introduction

Bone is a metabolically active tissue that undergoes constant remodeling. Human osteocalcin is a small, 49 amino acid protein produced by osteoblasts. Osteocalcin undergoes post-translational modification, in which three glutamic acid (Glu) residues, in positions 17, 21, and 24, convert to* γ-*carboxyglutamic acid (Gla) residues. This carboxylation process is vitamin K-dependent. Carboxylated osteocalcin (cOC) has a high affinity for Ca^2+^ and therefore it binds to bone mineral, whereas uncarboxylated (ucOC) form is unable to bind [1]. Both cOC and ucOC forms can be found in the circulation and circulating osteocalcin (total osteocalcin, tOC) consists also of truncated fragments and also partially carboxylated, or undercarboxylated forms [2, 3]. In humans, osteocalcin is incompletely carboxylated and uncarboxylation is more common at Glu17 than at two other positions [4, 5]. tOC measured from circulation is usually considered as a surrogate marker for bone formation [6]. In addition to biosynthesis in the osteoblasts, osteocalcin is also released from bone matrix during bone resorption [7] and thus, osteocalcin in circulation most likely reflects overall bone turnover.

Apart from being a marker for bone turnover, osteocalcin has been shown to participate as an endocrine factor in glucose and lipid metabolism. Osteocalcin-deficient mice have higher bone mineral density [8] but also increased body weight and impaired glucose metabolism [9]. Further studies indicated that the metabolic effect is due to ucOC form of osteocalcin, which has been shown to induce the secretion of insulin in pancreatic beta cells, both in mice [10] and in humans [11], adiponectin in adipocytes and to improve glucose uptake in skeletal muscle [9, 10, 12]. In particular, uncarboxylation at the first Glu residue (Glu17 in humans) has been linked to the endocrine effect in mice [13] and in humans [14]. The metabolic effect of ucOC is suggested to be mediated via G-protein-coupled receptor, GPRC6A [15, 16].

In humans, tOC levels have been shown to associate with circulating glucose levels, insulin sensitivity, and adiponectin concentration [17–20] in cross-sectional and observational studies which support the hypothesis of osteocalcin having an endocrine function. Meta-analyses have confirmed that tOC levels are lower in patients with type 2 diabetes (T2D) and that low tOC is a risk factor for T2D [21, 22]. On the contrary, Schwartz et al. reported no correlation between the incidence of diabetes and the use of antiresorptive therapy, which reduces circulating osteocalcin [23]. The association between the suggested hormonal form ucOC and glucose metabolism is less well understood. Low ucOC concentration has been shown to associate with T2D diagnosis [24]. Higher concentrations of ucOC are associated with enhanced insulin sensitivity and beta-cell function [25] but also correlate with better insulin secretion [26] as well as glycemic control and lower fasting plasma [27] in patients with diabetes. Other studies, however, have not been able to demonstrate a connection between ucOC and T2D [28] nor with insulin sensitivity [29] or insulin resistance [29, 30]. The evaluation of ucOC levels in human circulation has been done with different analytical methods, including direct ELISA [14] and hydroxyapatite binding assay [31], thus a reliable standardized method for measuring ucOC levels in circulation would be useful.

Recombinant antibody phage libraries provide a rapid alternative to immunization-based hybridoma technology for development of new monospecific antibodies for virtually any kinds of antigens. As antibody libraries are selected in vitro, the reaction conditions are easily adjusted to direct the selections, for example, towards specific post-translational modification sites [32]. In the current study, we developed recombinant antibodies that specifically detect ucOC form of osteocalcin, by using a synthetic single-chain fragment (scFv) antibody library [33]. We then developed a sensitive novel assay for the measurement of circulating ucOC in human blood samples and measured ucOC levels in carefully selected samples to get further insight to the association of ucOC and glucose metabolism in humans.

## Materials and Methods

### Antibodies, Assay Reagents, and Instruments

Monoclonal MAb-2H9 against human osteocalcin [34] was biotinylated with EZ-Link NHS-SS-PEG4-Biotin (Thermo Scientific, Rockford IL, USA). Polyclonal anti-*Escherichia coli* alkaline phosphatase (anti-AP, LS-C59288, LifeSpan Bioscience Inc., Seattle, WA, USA) was labeled with N1 europium chelate (Eu-anti-AP), as well as MAb-2H9, as described [35]. Human full-length ucOC peptide (1–49, Glu at positions 17, 21 and 24; C23–C29 bridge; purchased from Peptide 2.0, Chantivally, VA, USA) and cOC peptide (1–49; purchased from Sigma-Aldrich, Saint Louis, USA) were used and biotinylated. Microtiter wells coated with streptavidin (SA-plate), Buffer Solution RED (BSR), Wash Solution, and Europium Fluorescence Intensifier (EFI) were purchased from Kaivogen (Turku, Finland). All assay experiments were done in BSR, washing of the wells 4 times with wash solution and incubations at room temperature (RT) if not stated otherwise. Time-resolved fluorescence (TR-FIA) was enhanced by 200 µl EFI for 5 min and measured with Victor 1240 Multilabel Counter (Wallac/PerkinElmer Life Sciences, Waltham, MA, USA).

### Selection of ucOC-Specific Binders from Synthetic Antibody Library Using Phage Display

ucOC-specific binders were selected (panned) from the synthetic human scFv antibody libraries scFvM (diversity of 6 × 10^9^) and scFvP (diversity of 1 × 10^10^) using phage-display technique, as described [33, 36]. In brief, panning was done in three rounds of positive selection against bio-ucOC on paramagnetic streptavidin beads using cOC as a free blocker. Bound phages were eluted with trypsin. *E. coli* XL1-Blue cells were infected with the eluted phages and new phage stocks were produced with VCS M13 helper phage. In a parallel experiment, selection against bio-ucOC and bio-cOC (without blocker) was alternated in different panning rounds to isolate binders recognizing both forms of osteocalcin (Supplemental data). Individual clones were isolated from the enriched libraries as scFv fused with bacterial alkaline phosphatase (scFv-AP) and screened for binding to bio-ucOC.

Based on the ucOC-specificity of soluble scFv-AP fusion proteins, the best clones were targeted for mutagenesis and further panning in order to obtain modified antibodies with improved binding. To produce the mutagenesis libraries, sequence, and length variation was introduced in CDR loops L1, L3, H1, and H2 using oligonucleotide-directed mutagenesis [37]. The new scFv phage libraries were combined and subjected to two rounds of panning against ucOC followed by screening of individual clones for binding to ucOC and cOC. Selected candidates were cloned in pLK06H vector [36] and produced in *E. coli* as soluble scFv-AP proteins (Supplemental data).

### Production and Purification of Recombinant Antibody Fragments

Selected scFv clones were converted to human IgG1,κ Fab. Synthetic human Fab genes were purchased from GeneArt (Thermo Scientific) and cloned at SfiI sites into vector pLK06FT, which is similar to the vector pLK06H [33], by providing resistance for ampicillin, bacterial AP fusion protein with His6 but also contains a FLAG tag. Both scFv pLK06H and Fab pLK06FT-containing vectors were transformed into *E. coli* BL21 cells for antibody production. The production was done as a shake flask culture (*V* = 200 ml) and antibody expressions were induced for 10 h with IPTG at 22–26 °C. After collection, purification of the antibodies was done with Ni–NTA affinity chromatography (HisPur, Thermo Scientific) according to the manufacturer’s instructions. Concentrations were measured at 280 nm (NanoDrop1000 spectrophotometer, Thermo Scientific) and protein purity was analyzed with SDS-page (data not shown).

### Antibody Characterization

Thermostability of the recombinant antibodies was measured by heating in the presence of 5 mM DTT (Thermo Scientific), at temperatures ranging from 40 to 71 °C for 60 min. After warming, immunoreactivity was assessed.

Antibody binding site on human osteocalcin was determined using BioTides™ Biotinylated Peptides (JPT Peptide Technologies, Germany). Peptides with length of 22 amino acids and offset of one amino acid were designed to cover the osteocalcin sequence. To determine the binding of Fab-APs at each carboxylation site, we used three biotinylated peptides corresponding to Leu6-Leu25 and harboring only one carboxylation (Gla) at position 17 or 21 or 24. In addition, we used a fully carboxylated Leu6-Leu25 peptide with carboxylation at all three carboxylation sites (Gla17 and Gla21 and Gla24). In brief, SA-plates were coated with BioTide peptides, Fab-AP-antibodies were added and finally, Eu-anti-AP was used for detection by TR-FIA measurement.

EC50 values were determined using SA-wells coated with 1.4 pM of bio-ucOC, followed by recombinant antibodies and either Eu-anti-AP or Eu-MAb-2H9.

Antibodies were used in immunofluorescence staining for osteocalcin in osteoblasts differentiated from rat mesenchymal stromal cells (Supplemental data). In addition, the culture medium of the treated cells was analyzed for ucOC.

### Assay for ucOC and Characterization

Recombinant antibodies were used to develop a two-site immunoassay. SA-plates were coated with bio-MAb-2H9 (200 ng/well). Next, 100 µl of standard or sample with 100 µl BSR containing 5 mM EDTA was added for 60 min. Fab-AP and Eu-anti-AP (100 ng/well) were added for 90 min. Finally, EFI was added and TR-FIA was measured. Assay total volume was 200 µl and ucOC peptide in BSR (range 0.11–9 ng/ml) was used as a standard. Signals shown were background corrected. Logarithmic variants of detected signals were used to evaluate the samples.

Assay performance was optimized for Fab-AP concentration (50 to 365 ng/well), incubation time (30 to 120 min), assay volumes (50 to 200 µl), and assay schemes, and preliminary characterization of the assay was performed. Cross-reactivity to cOC was determined. Assay linearity and assay matrix affect were analyzed by adding 8 ng/ml of ucOC peptide either in BSR or biological sample and performing serial dilutions in the same matrix. Within-run variability was defined by 8 replicate samples and total variability by 36 plasma samples. Between-run variability was measured with two plasma samples in four different runs. The lowest limit of detection (LOD) was calculated from three standard deviations (SD) over the background (*N* = 11) and the lowest limit of quantification (LOQ) from diluted plasma sample with ucOC. Novel immunoassay was compared with an ELISA assay for ucOC (Glu-OC EIA kit, Takara Bio Inc, Japan) using serum (*N* = 20), heparin plasma (*N* = 20) and EDTA plasma (*N* = 20) samples, according to the manufacturer’s instructions.

### Blood Samples

Blood samples from 14 healthy volunteers (7 males and 7 females, age 22–48 years, mean 28.1 ± 6.5 years) were collected at multiple times after 10–12 h overnight fast in collection tubes (Vacuette®, Greiner) for serum (with or without gel), (lithium) heparin plasma or EDTA plasma (with EDTA K2) according to manufacturer’s instruction. Samples were transferred on ice, aliquoted, and stored at − 80 °C within 60 min from sample collection. In addition, serum and EDTA plasma samples were collected simultaneously from three volunteers, aliquoted and incubated at + 4 °C, RT (+ 22 °C) or at − 20 °C to assess in vitro stability of ucOC. The effect of freeze–thaw cycles (5 times) was also tested. Samples were transferred at − 80 °C and a reference sample was stored at − 80 °C immediately after collection.

Blood sampling was approved by the local ethical committee and samples were collected in accordance with the Helsinki Declaration. Informed consent was obtained from all participants.

### EDTA Plasma Samples from Biobank

Aliquots of human EDTA plasma were from Auria Biobank, Turku, Finland. Samples had been collected from volunteer donors and stored at − 80 °C. We identified 49 patients with confirmed T2D diagnosis (ICD10 E11). Inclusion criteria were as follows: fasting plasma sample available (overnight fast), result of fP-Glucose measurement, and sample stored at − 80 °C within six hours from sampling. 46 age- and sex-matched controls without T2D and with normal fP-Glucose (Con-NG, fP-Glucose ≤ 6 mmol/l) were identified from biobank. In addition, a second set of age - and sex-matched controls were selected. These subjects did not have confirmed T2D diagnosis but had high fP-Glucose (Con-HG, fP-Glucose ≥ 7 mmol/l, *N* = 29). These could represent potentially undiagnosed T2D patients. Exclusion criteria were as follows: any cancer, fracture within one year from sampling or the use of bone-active medication (bisphosphonates, denosumab, teriparatide). Samples were analyzed for ucOC, as described. tOC (methods in supplemental data) and N-terminal propeptides of type I collagen (IDS-iSYS Intact PINP, IDS, UK) were measured as a marker of bone formation.

### Statistical Analysis

Data are presented as mean with standard deviations or as medians with interquartile range. The distribution of the variables was evaluated using Shapiro–Wilk test. ucOC, tOC, fP-Glucose, and PINP were not normally distributed (Shapiro–Wilk < 0.95) and logarithmic transformations (ucOC, tOC, fP-Glucose, PINP) were used for analyses. One-way ANOVA was used to test differences between groups. Spearman’s tests (non-normally distributed data) were used to study the correlations between variables. SPSS, version 25 (SPSS, Inc., Chicago, IL, USA), was used for data analysis.

## Results

### New ucOC-Specific Binders were Isolated by Phage Display from Synthetic Antibody Libraries

UcOC-specific binders were selected from the synthetic human scFv antibody libraries scFvM and scFvP by phage-display technique. ScFv-AP13 was one of the clones isolated in the panning against bio-ucOC and cOC as a free blocker. Clones scFv-AP2, scFv-AP16, and scFv-AP19 present mutated variants of scFv-AP13 isolated from a secondary libraries built by rerandomizing either CDR-L1, L3, H1, and/or H2 loops of scFv-AP13. These four antibodies shared an identical CDR-H3 but had sequence and loop length variation in loops CDR-H2 (scFv-AP2 and scFv-AP19), and CDR-H1 (scFv-AP16) (data not shown).

### Stability of ucOC-Specific Recombinant Antibodies was Improved by scFv to Fab Conversion

**Fig. 1:**
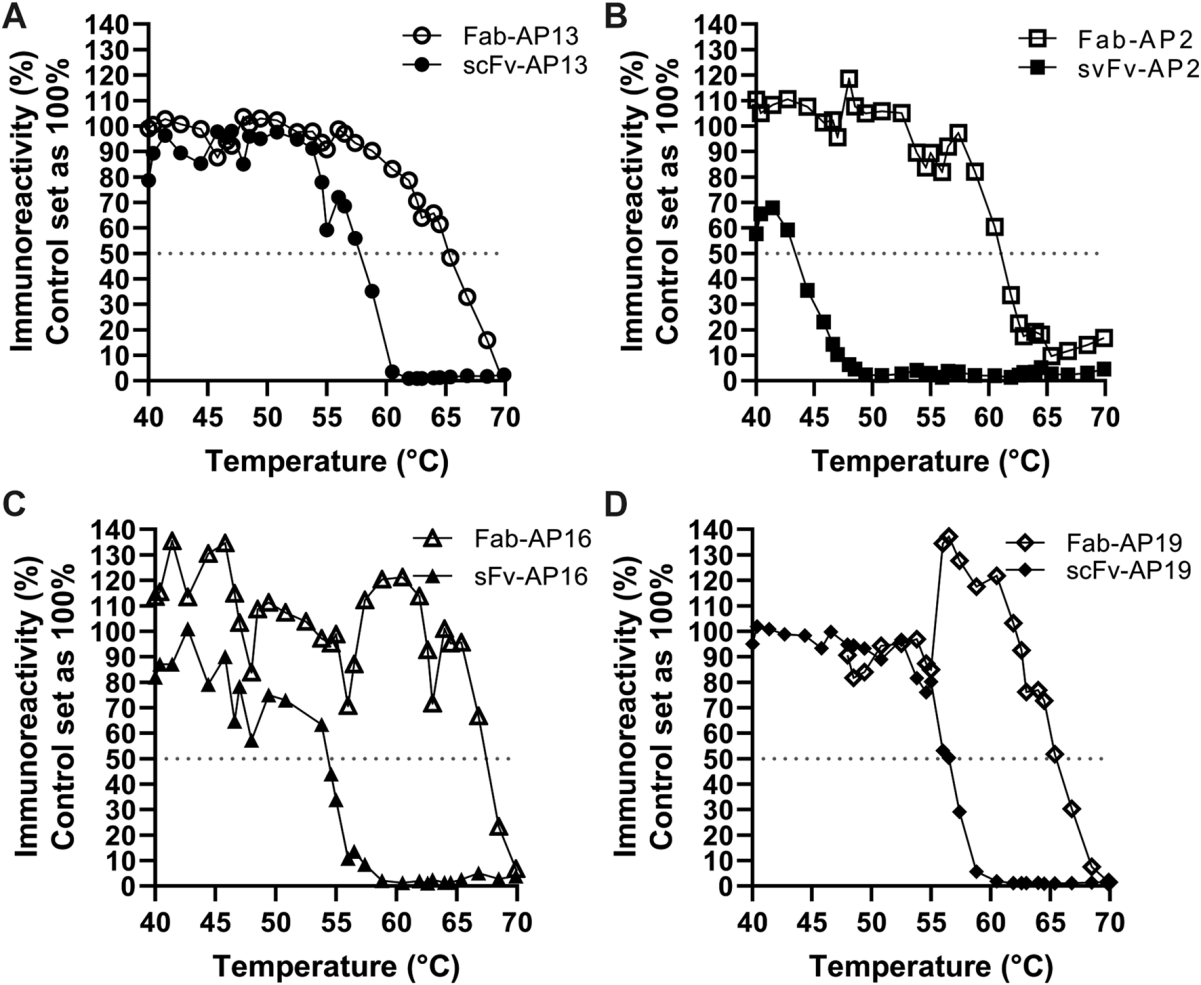
Conversion from scFv-AP to Fab-AP improved the thermostability of the antibodies. Thermostability of the recombinant antibodies Fab-AP (open) and scFv-AP (solid) was tested by heating the antibodies Fab-AP13 (**a**), Fab-AP2 (**b**), Fab-AP16 (**c**), and Fab-AP19 (**d**) from 40 °C up to 71 °C. Reference antibody was kept on ice during the heating process and set as 100% and Tm was assessed when 50% of immunoreactivity was lost (gray dot line)

The scFv antibodies were converted into Fab-AP fragments in order to improve stability. Thermostability was evaluated by heating the recombinant antibodies and measuring the temperature (Tm) where 50% of immunoreactivity was lost when compared to unheated control. Tm of the Fab fragments varied between 60 °C and 66 °C. ScFv to Fab conversion improved the thermostability of antibodies Fab-AP13 (Fig. 1a), Fab-AP16 (Fig. 1c), and Fab-AP19 (Fig. 1d) by approximately 10 °C. The most increase in stability, approximately 16 °C, was observed in the scFv-AP2 to Fab-AP2 conversion (Fig. 1b).

### ucOC-Specific Fabs Recognize Glu17 or Glu 21 in Human ucOC

**Fig. 2:**
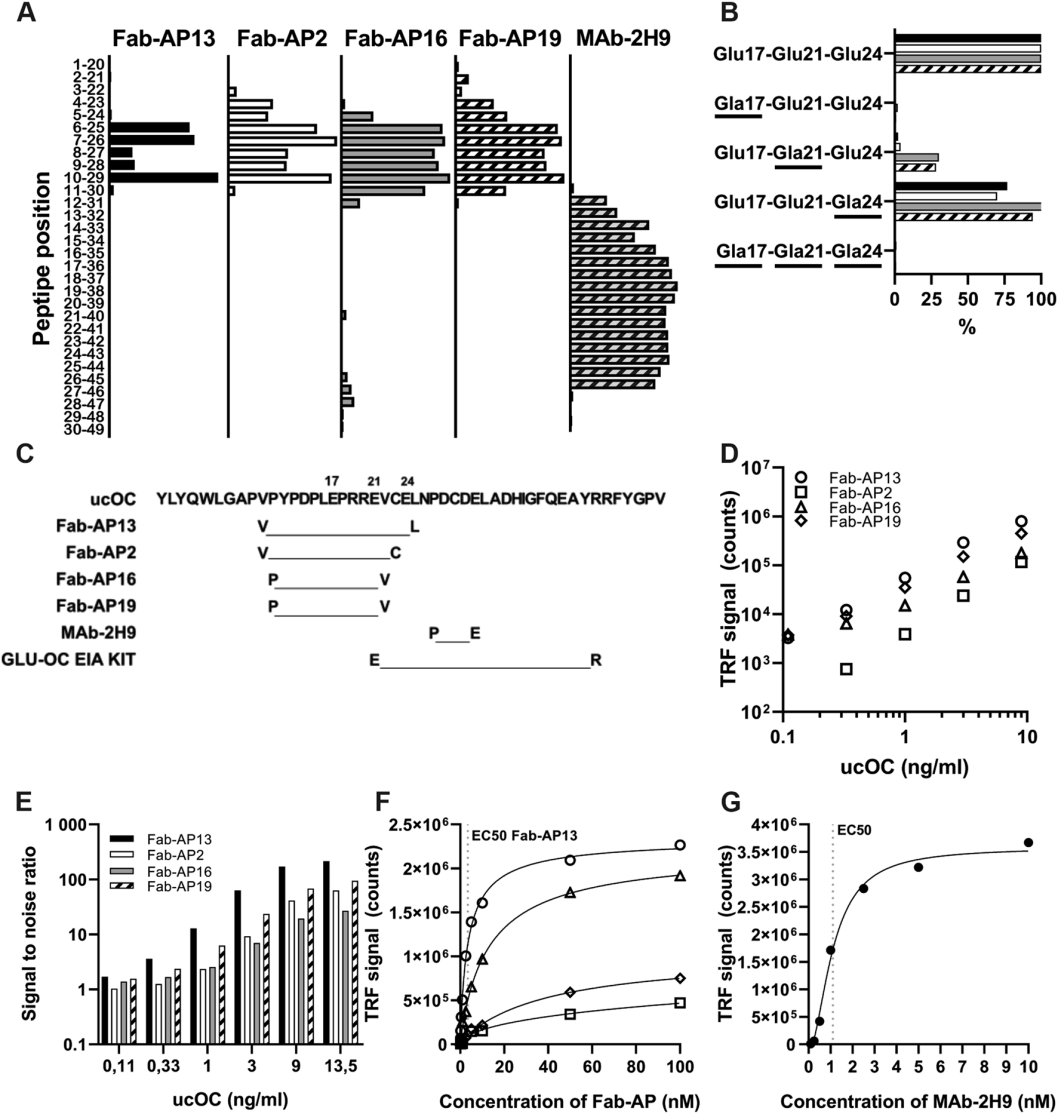
Characterization of the recombinant Fab-AP–antibodies. The binding site in ucOC was determined for Fab-AP13 (black), Fab-AP2 (white), Fab-AP16 (gray), Fab-AP19 (white with stripes) and MAb-2H9 (gray with stripes) by testing biotinylated linear human osteocalcin peptides (**a**). In addition, carboxylation specificity was determined by linear peptides with different carboxylation status in positions aa17, aa21, and aa24 (**b**). Overview on the epitopes of the antibodies (incl. commercial Glu-OC EIA kit) on ucOC sequence 1–49 (**c**). N and C-terminal residues of the epitopes are indicated. The epitope of Glu-OC EIA kit is based on the information provided in kit insert. Time-resolved fluorescence signals (**d**) and signal-to-noise ratios (**e**) in a two-site immunoassay obtained for Fab-AP13 (circle), Fab-AP2 (square), Fab-AP16 (triangle), and Fab-AP19 (diamond) with MAb-2H9 as capture and ucOC peptide concentrations 0.11–13.5 ng/ml). EC50 values for recombinant antibodies binding to bio-ucOC (**f**) were determined by diluting the antibody from 100 nM to 0.001 nM. EC50 value was also determined for MAb-2H9 (**g**) by diluting the antibody from 10 nM to 0,001 nM. Note that the* x* axis is different in figures F and G

The epitopes of the antibodies and their specificity to carboxylation of ucOC were determined. The binding site of Fab-AP13 was concluded to be amino acids Val10-Leu25 and for Fab-AP2 amino acids Val10-Cys23. The binding sites of Fab-AP16 and Fab-AP19 were slightly more C-terminal by one residue, at amino acids Pro11-Val22 (Fig. 2a). At carboxylation site, binding of Fab-AP13 and Fab-AP2 was dependent on Glu residues at positions Glu17 and Glu21, while residue at position 24 could be either Glu or Gla. Both Fab-AP16 and Fab-AP19 were dependent on Glu17 only (Fig. 2b). The binding site of MAb-2H9 was tested as well and was found to reside at C-terminal part of the osteocalcin peptide, at Pro27-Glu31 (Fig. 2a), and not containing residues undergoing carboxylation. Hence, it recognizes both ucOC and cOC similarly. The epitopes are summarized in Fig. 2c.

### New Antibodies have Different Characteristics and Detect Endogenous ucOC

MAb-2H9 was used as a capture and either Fab-AP13, -2, -16, or -19 as detecting antibody along with Eu-anti-AP to measure time-resolved fluorometry in the immunoassay.

The optimal amount of each Fab-AP antibody used in the assay was determined by highest signal-to-background ratio. Fab-AP13, Fab-AP2, and Fab-AP19 achieved the best assay sensitivity with 100 ng/well and Fab-AP16 with 50 ng/well (data not shown). The four antibodies were compared by measuring up to 13.5 ng/ml concentration of synthetic ucOC peptide. The signal levels obtained with different antibodies varied: Fab-AP13 showed highest signal, being approximately 1*10^6^ counts at the highest ucOC concentration. Second highest signal was detected with Fab-AP19 (0.6*10^6^ counts), while Fab-AP16 and Fab-AP2 had lowest maximum signals (0.3*10^6^ and 0.2*10^6^ counts, respectively) (Fig. 2d). Fab-AP13, -16, and -19 were able to detect the lowest concentration of the calibrator, 0.11 ng/ml, but Fab-AP2 was not (Fig. 2d). Fab-AP13 had highest signal-to-noise ratio in all tested ucOC concentrations. Although Fab-AP16 had higher absolute signal compared to Fab-AP2, signal-to-noise ratio of Fab-AP16 was lower (Fig. 2e). EC50 was determined with four parameter variable slope. EC50 value for Fab-AP13 binding to bio-ucOC was 3.5 nM. for Fab-AP16 it was 13.2 nM and for Fab-AP19 value was 39.7 nM. EC50 value for Fab-AP2 was undetectable in this range, as no saturation of the curve was observed (Fig. 2f). In addition, we analyzed EC50 value for the capture antibody MAb-2H9, which resulted in a value of 1.1 nM (Fig. 2g).

**Fig. 3:**
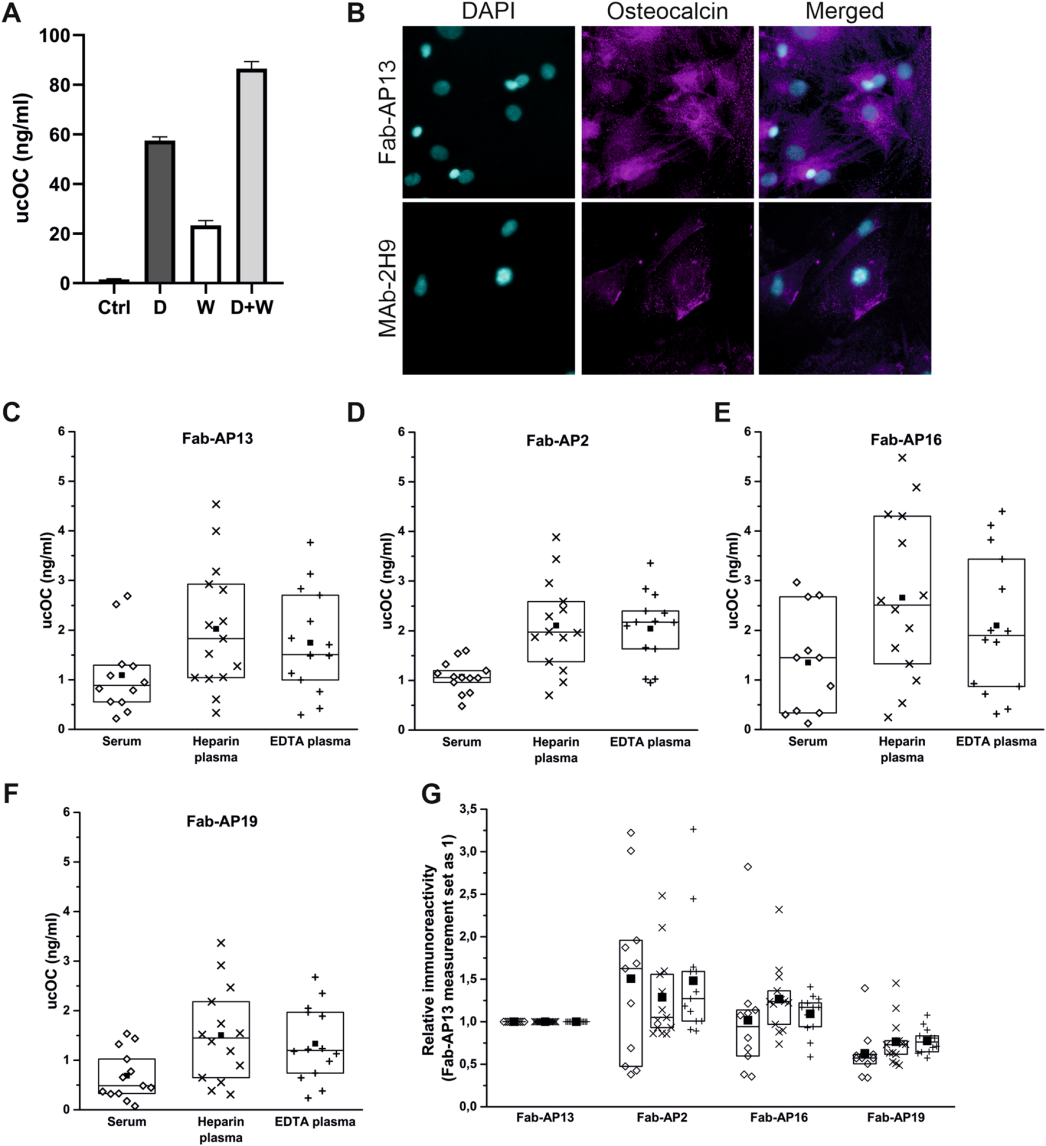
Recombinant antibodies detect biosynthetic ucOC. Biosynthetic ucOC was measured from the medium of rat osteoblasts cultured for 9 days (**a**; without treatment black; vitamin D gray; warfarin white and vitamin D along with warfarin light gray). In addition, Fab-AP13 and MAb-2H9 were used in immunostaining of ucOC and tOC, respectively (**b**). Serum, heparin, and EDTA plasma samples (*N* = 14) were measured with Fab-AP13 (**c**), Fab-AP2 (**d**), Fab-AP16 (**e**) and Fab-AP19 (**f**) immunoassays and concentrations of detected ucOC are shown. In figure **g**, relative immunoreactivity is shown and immunoassay based on Fab-AP13 is set as 1 and other measurements of immunoassays compared. Serum is shown as diamond, heparin plasma as cross and EDTA plasma as plus signs. The lines of the boxes represent the 25th, 50th, and 75th percentiles and the black square indicates the mean value

To test whether our immunoassay measures biosynthetic ucOC, osteocalcin expression was upregulated with 1,25(OH)_2_ dihydroxy vitamin D (vitamin D) in cultured rat osteoblasts and ucOC secreted into medium was measured. Warfarin treatment was used to inhibit carboxylation of osteocalcin. Treatment with warfarin and vitamin D increased detectable ucOC by approximately 50% when compared to cells treated with vitamin D alone (Fig. 3a). This suggests that novel immunoassay measured biosynthetic ucOC derived from osteoblasts. In addition, Fab-AP13 and MAb-2H9 detected biosynthetic ucOC and tOC, respectively, in cultured rat osteoblasts, as indicated by immunofluorescence staining (Fig. 3b).

To compare the antibody performance in different sample matrices, ucOC levels were measured in serum, heparin plasma, and EDTA plasma samples using varying Fab-AP for detection. All antibodies detected endogenous ucOC in blood samples. There were no significant differences between serum samples with or without gel (data not shown). All measured ucOC concentrations were higher in plasma samples when compared to paired serum samples. The mean values for heparin and EDTA plasma samples were on average 131% (range 97–179%) and 73% (range 60–113%) higher, respectively, than in serum, depending on the antibody (Fig. 3c–f).

When immunoreactivities detected by different antibodies were compared to Fab-AP13-based immunoassay, we observed that ucOC levels measured by Fab-AP13 and Fab-AP16 in paired samples were relatively similar, showing difference of 1.0-fold (serum), 1.3-fold (heparin plasma), and 1.1-fold (EDTA plasma), respectively (Fig. 3g). Instead, ucOC levels measured by Fab-AP2 based assay were higher by 1.5-fold, 1.3-fold, and 1.5-fold, respectively, and assay based on Fab-AP19 appeared to underestimate ucOC levels (0.6-fold, 0.8-fold, and 0.8-fold, respectively) (Fig. 3g).

### Fab-AP13-Based Immunoassay Detects ucOC in Human Serum and Plasma

**Fig. 4:**
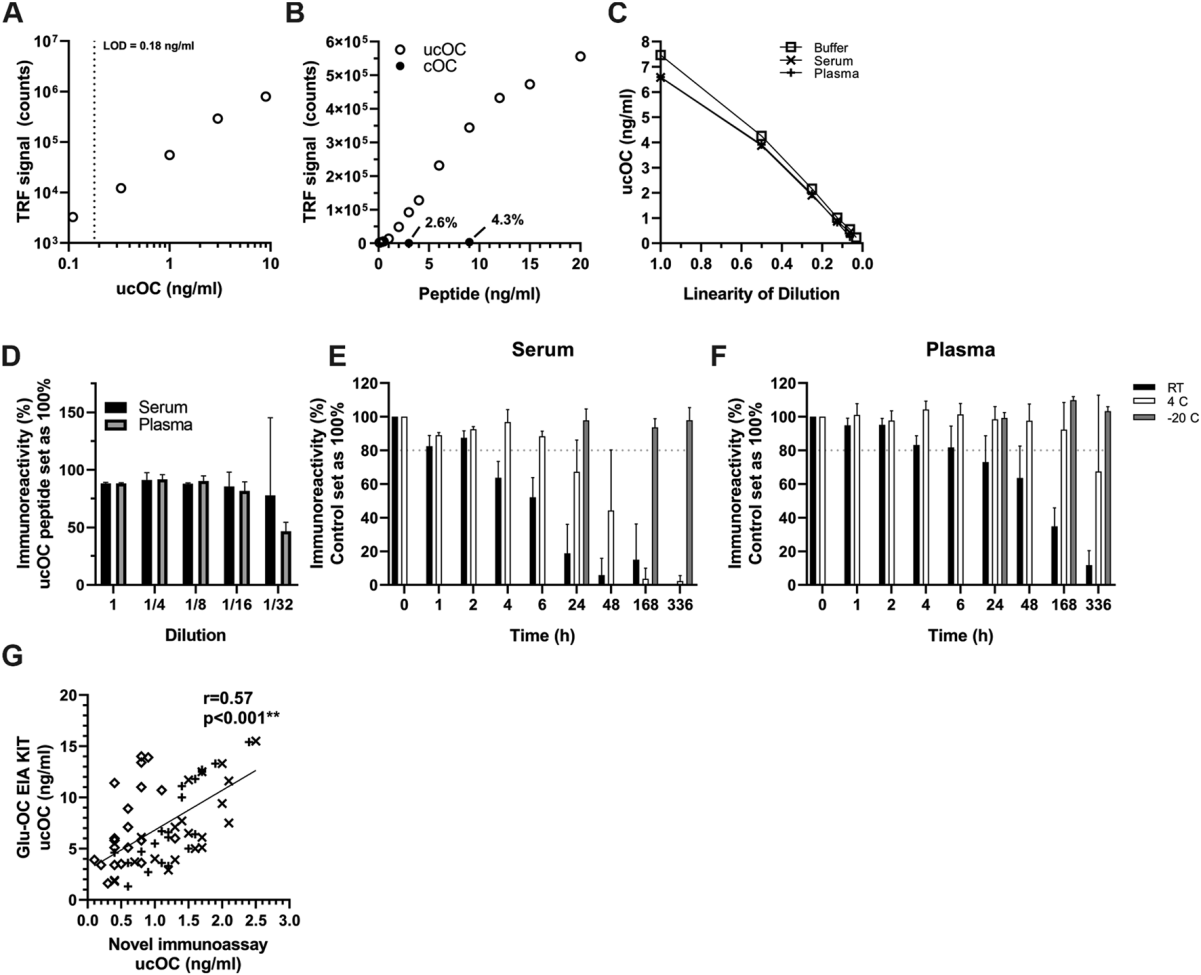
Characterization of immunoassay based on Fab-AP13. Representative figure of standard curve is presented (**a**). Cross-reactivity to cOC of Fab-AP13-based immunoassay was determined by measuring cOC peptide (solid circles) from 1 ng/ml up to concentration of 1000 ng/ml and cross-referencing signal to measurement of ucOC peptide (open circles) at the same time (**b**). 8 ng/ml ucOC peptide, spiked in either BSR (square), serum (cross), or plasma (plus sign), was diluted in BSR and linearity was observed (**c**). 8 ng/ml of ucOC peptide spiked in serum (black) and plasma (gray) samples was measured by diluting samples (**d**). Undiluted sample of 8 ng/ml was set as 100%. In vitro stability of ucOC in serum (**e**) and plasma (**f**) was assessed. Samples (*N* = 3) were stored for indicated times at + 22 °C (black), + 4 °C (white) or at − 20 °C (gray). Reference sample was stored at − 80 °C and set as 100%. Fab-AP13-based immunoassay was compared to Glu-OC EIA kit with 60 blood samples (serum presented as diamond, *N* = 20; heparin plasma as plus sign, *N* = 20; EDTA plasma as cross, *N* = 20) and Spearman’s correlation was determined (**g**). Note that the scale on* x* axis is different

Based on the epitope, thermostability, and performance with peptide and blood samples, we continued with Fab-AP13 for immunoassay optimization. Representative standard curve is shown in Fig. 4a. LOD was determined to be 0.18 ng/ml, between-run variability 8.5%, within-run variability 6.1% and total variability 10.7%. Cross-reactivity to cOC was 2.3% at 3 ng/ml, 4.3% at 9 ng/ml and 0.2% at the excess of 1000 ng/ml cOC peptide (Fig. 4b). With the acceptance of 20% deviation from the expected value, ucOC peptide spiked in either serum and in plasma was linear up to dilution 0.125, which equals to 0.87 ng/ml and 0.83 ng/ml ucOC, respectively (Fig. 4c). Immunoreactivity measured in serum and plasma samples spiked with ucOC were on average 12% lower than spiked buffer samples (Fig. 4d).

Storage of serum and plasma samples at RT significantly decreased the stability of endogenous ucOC. UcOC concentration detected in serum samples rapidly decreased to 87%, 64%, and 52% by 2, 4, and 6 h incubation at RT, respectively. Immunoreactivity was preserved at + 4 °C, as there was still 93%, 97% and 88% immunoreactivity remaining at 2, 4, and 6 h (Fig. 4f). ucOC appeared to be more stable in plasma samples, as immunoreactivity was 83% after 4 h and 73% after 24 h at RT. At + 4 °C, immunoreactivity remained at 98% after 24 h and decreased to 64% after 48 h storage. Immunoreactivity was not lost in samples stored at – 20 °C within the time period analyzed (Fig. 4g) nor when samples underwent repeated freeze–thaw cycling (data not shown). An arbitrary cut-off for acceptable loss of immunoreactivity was set at 20%. In serum, immunoreactivity decreased below 80% of control between 2 and 4 h at RT and after 6 but before 24 h at + 4 °C In plasma, the corresponding times were between 6 and 24 h at RT and 1 and 2 weeks at + 4 °C.

Fab-AP13-based immunoassay was compared to a commercial Glu-OC EIA kit by analyzing 60 blood samples in both assays. Levels of ucOC were on average 6.5-fold higher when measured with Glu-OC EIA kit compared to Fab-AP13 immunoassay, but the results were significantly correlated in all samples (*r* = 0.57, *p* < 0.001, *N* = 60) (Fig. 4g). The association was observed in all sample matrices, but it was less pronounced in serum (*N* = 20, *p* = 0.004) than in EDTA plasma (*N* = 20, *p* < 0.001) and heparin plasma (*N* = 20, *p* < 0.001).

### ucOC Levels Associate with Plasma Glucose Levels, but not with T2D Diagnosis

**Fig. 5:**
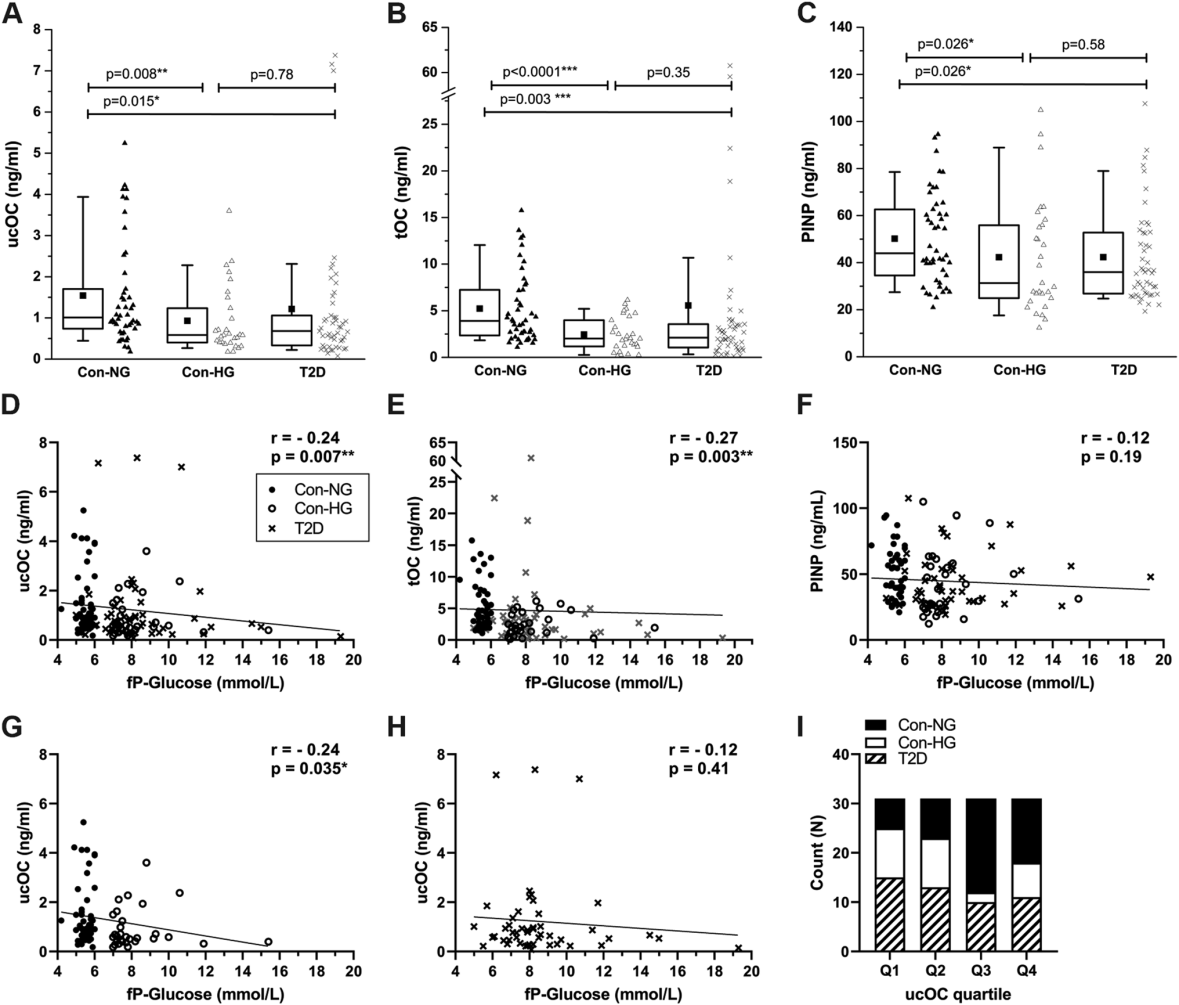
ucOC correlated with plasma glucose levels in non-diabetic subjects. Measured levels of ucOC (**a**), tOC (**b**), and PINP (**c**) are presented. Normoglycemic controls (Con-NG, *N* = 46) are shown as triangles, hyperglycemic controls (Con-HG, *N* = 29) are shown as open triangles and T2D patients (T2D, *N* = 49) as cross and *p*-values for one-way ANOVA are shown. Correlation between ucOC levels (**d**), tOC levels (**e**), and PINP levels (**f**) with fP-Glucose in all three groups combined are shown. Correlation between ucOC levels and fP-Glucose of control groups (**g**) and T2D group (**h**) is shown. Correlation was determined by Spearman’s test and Con-NG is shown as circle, Con-HG is shown as open circle and T2D is shown as cross. Samples were categorized as quartiles (**i**; Q1–Q4) and Con-NG shown as black bar, Con-HG shown as white bar and T2D shown as white bar with stripes

Table 1 summarizes the characteristics of subjects. ucOC levels were significantly lower in subjects that had hyperglycemia (Con-HG, median ucOC 0.58 ng/ml, *p* = 0.008) than in subjects with normal plasma glucose, i.e., ≤ 6 mmol/l (Con-NG, median 1.01 ng/ml). Similarly, patients with T2D diagnosis had significantly lower ucOC levels (T2D, median 0.68 ng/mL, *p* = 0.015) than normoglycemic controls. There was no difference between subjects with hyperglycemia and subjects with T2D diagnosis (*p* = 0.72) (Fig. 5a). Con-HG group also had significantly lower tOC levels (median 2.01 ng/ml, *p* < 0.001) compared to Con-NG (median 3.90 ng/ml). T2D group also had significantly lower tOC levels (median 2.11 ng/ml, *p* = 0.003) than normoglycemic controls (Fig. 5b). The trend was similar for PINP but the differences between the groups were not as pronounced as for ucOC and tOC. Con-HG group had significantly lower PINP levels (median 31.3 ng/ml, *p* = 0.026) compared to Con-NG group (median 44.0 ng/ml). Similarly, T2D group had significantly lower levels (median 36.0 ng/ml, *p* = 0.026) compared to Con-NG group (Fig. 5c).

**Table 1:** Characteristics of subjects (*N* = 124)

	Con-NG	Con-HG	T2D	*p* value
*N*	46	29	49	
M/F (*N*)	23/23	13/16	24/25	0.90
Age (years)	66.3 (9.6)	64.5 (9.1)	65.7 (9.6)	0.70
fP-Glucose (mmol/l)	5.50 (5.3–5.8)	7.70 (7.2–8.7)	8.00 (7.1–9.1)	** < 0.001**
ucOC (ng/ml)	1.01 (0.72–1.80)	0.58 (0.40–1.47)	0.68 (0.32–1.21)	**0.015**
tOC (ng/ml)	3.90 (2.31–7.38)	2.01 (1.15–4.20)	2.11 (1.02–3.70)	**0.001**
PINP (ng/ml)	44.0 (34.0–63.2)	31.3 (24.2–57.1)	36.0 (26.7–52.9)	**0.036**
Osteoporosis^a^ (*N*)	3	1	3	0.84
Use of medications^b^				
Anti-diabetic^c^ (*N*)	0	0	19	** < 0.001**
Estrogens (*N*)	5	1	2	0.31
Cortisone (*N*)	0	0	3	0.10
Warfarin (*N*)	0	1	1	0.49

Bold values indicate statistical significance (*p*  < 0.05)

EDTA plasma samples were obtained from normoglycemic (NG) and hyperglycemic (HG) subjects and patients with type 2 diabetes (T2D). Data are presented as mean with SD (age) or median with interquartile range (fP-Glucose, ucOC, tOC and PINP) or number of subjects (gender, medications). One-way ANOVA (for fP-Glucose, ucOC, tOC and PINP after log-transformation) or *χ*^2^ test were used to test differences across the groups and statistically significant differences are highlighted

^a^Diagnosis for osteoporosis (ICD10 code M80 or M81)

^b^None of the subjects were on bone-active medication (bisphosphonates, denosumab, teriparatide)

^c^Anti-diabetic medications were as follows: insulin, metformin, sitagliptin, liraglutide, or gliflozins

ucOC levels (Fig. 5d) were negatively correlated with fP-Glucose (*r* = − 0.24, *p* = 0.007) in all samples. Negative correlation was also seen for tOC and fP-Glucose (*r* = − 0.27, *p* = 0.003) (Fig. 5e). Interestingly, PINP levels did not correlate with fP-Glucose (*r* = − 0.12, *p* = 0.19) (Fig. 5f). The correlation between ucOC and fP-Glucose levels was significant particularly in subjects without T2D diagnosis (*r* = − 0.24, *p* = 0.035) (Fig. 5g) but not in T2D group (*r* = − 0.12, *p* = 0.41) (Fig. 5h). Subjects were further divided into quartiles (*N* = 31 each) according to the plasma ucOC levels. Con-NG group was highly represented (69.6%) in higher quartile groups (Q3 + Q4). Interestingly, Con-HG subjects were mainly (70.0%) found in the lower ucOC quartiles (Q1 + Q2). T2D subjects were equally distributed between quartiles (Fig. 5i).

## Discussion

In this study, we developed stable and high-affinity ucOC-specific recombinant antibodies and a highly sensitive pilot immunoassay to detect ucOC levels from serum and plasma samples in human. In addition, the new antibodies described in this study can be used as a tool in biomedical research applications, such as immunofluorescence staining. We used single-framework antibody phage libraries to construct four specific recombinant antibodies that were thoroughly characterized. The antibody Fab-AP13 with the best performance was selected and used in the final, characterized immunoassay. With the established immunoassay, we measured ucOC levels in human plasma samples and discovered that ucOC levels negatively correlated with glucose levels in non-diabetic adults. Based on antibody characterization, our assay detects fully uncarboxylated ucOC but also partially carboxylated osteocalcin with Glu at positions 17 and 21. In addition to full-length ucOC, the assay detects also ucOC fragments containing mid-molecular region Val10-Glu31 and uncarboxylation at Glu17 and Glu21.

All four studied antibodies recognized the same region in ucOC. Their epitope was located in the N-terminal half of the molecule and uncarboxylation at amino acid Glu17 was required for all of them to detect ucOC. This is expected as the antibodies included a primary library derived Fab-AP13 and its three further mutated variants Fab-AP2, -13 and -19, all of which had been selected for binding to ucOC. Moreover, the antibodies shared identical CDR-H3 loop which is the major determinant of antibody specificity. The antibodies still show differences in terms of their capability binding ucOC, and interestingly also in their fine-specificity, which properties can be attributed to mutations found in either CDR-L1 or CDR-H2 loops. Fab-AP13 and Fab-AP2 antibodies preferred uncarboxylation at amino acid Glu21 but on the contrary, Fab-AP16 and Fab-AP19 did not. When comparing the novel immunoassays based on these antibodies, we used Fab-AP13 as a reference antibody. Fab-AP2 seemed to detect endogenous ucOC over the peptide. The ucOC levels on samples measured by Fab-AP2 were found to be higher than measured with Fab-AP13 but the signal levels of the standard, on the contrary, were found to be only a fifth of the signal of Fab-AP13. Interestingly, Fab-AP19 detected lower levels of endogenous ucOC than Fab-AP13. The absolute signals of the standards also almost half lower compared to Fab-AP13. These could suggest that Fab-AP19 prefers the peptide over endogenous ucOC. ucOC levels detected by Fab-AP16 were similar to those by Fab-AP13, but the measured signals were much lower. EC50 values strongly indicated that Fab-AP13 is the most feasible antibody for the immunoassay: The EC50 value for Fab-AP13 was 3.5 nM and the second best was Fab-AP16 with value of 13.2 nM.

Our novel immunoassay, based on the antibody Fab-AP13, was characterized and found to be sensitive with LOD 0.18 ng/ml and biological sample linear down to dilution 0.8 ng/ml in serum and in plasma samples. When biological samples were spiked with ucOC peptide, immunoreactivity measured in the spiked serum and plasma samples were 10% lower compared to their buffer counter. This suggests that the biological samples could either have degrading proteases or serum and plasma matrices slightly interfere with the antibody binding. ucOC level detected by Fab-AP13 immunoassay degraded rapidly when serum samples were kept at RT. Rapid degradation of intact osteocalcin in serum has been reported previously [3]. The stability of immunoreactivity was significantly improved at + 4 °C and no loss of immunoreactivity was observed when samples were kept at − 20 °C. ucOC immunoreactivity was more stable in plasma than in serum. The detection of the analyte, ucOC, from biological sample depends on the antibody binding site and its stability. Fab-AP13 binds to more stable epitopes of osteocalcin. With our novel immunoassay, ucOC form of osteocalcin can be reliably detected in serum and plasma samples stored at − 20 °C or + 4 °C. Immunoreactivity is preserved at RT for 24 h in EDTA plasma samples and for 6 h in serum samples. We also analyzed 60 blood samples with both Fab-AP13 based immunoassay and commercial Glu-OC EIA kit. ucOC concentrations measured by our immunoassay were lower, most like due to the differences in assay calibration, but the measurements were well correlated between the two assays. Correlation was observed particularly in plasma samples. Our Fab-AP13-based assay measures fully uncarboxylated ucOC but detects also partially uncarboxylated osteocalcin with Glu at position 17, while Takara’s Glu-OC EIA kit measures only fully uncarboxylated ucOC [14]. This could explain some the differences.

With the immunoassay developed in this study, we measured a pilot study with adult subjects with either normal fasting glucose, elevated fasting glucose but no confirmed T2D diagnosis, and also patients with diagnosed T2D. Samples were selected from a collection of archived plasma samples (biobank) and carefully defined inclusion and exclusion criteria were applied. Patients with T2D had lower concentrations of ucOC compared to the normoglycemic control group. ucOC levels in subjects with elevated glucose levels were closer to the T2D patients. This has been reported previously also for tOC [17]. In subjects without T2D, ucOC levels negatively correlated with fasting plasma glucose levels, but this correlation was not found in patients with T2D. Similar association was found for blood glucose and tOC levels. In this study, ucOC-specific assay did not provide major additive value over tOC, probably because ucOC is a fraction of tOC and thus tOC measurement also captures changes in ucOC. Interestingly, similar correlation to glucose levels was not observed with type I collagen propeptides, PINP. PINP and osteocalcin are both secreted by the osteoblasts and used as markers of bone formation [6]. This could indicate that plasma glucose is associated more specifically to osteocalcin protein, in particular ucOC, but not to bone formation rate in general, or that, in addition to biosynthesis, ucOC is also released during bone resorption. We then classified all the subjects into quartiles according to their ucOC level, and in line with correlation analysis, found that individuals with hyperglycemia (≥ 7 mmol/l) were more frequently found in the lower ucOC quartiles and, accordingly, individuals with normal glucose levels were predominantly present in the higher ucOC quartiles. Patients with T2D were equally distributed throughout the quartiles. The lack of association between ucOC levels and T2D diagnosis in our study is in contrast to some previous reports [28–30]. This could be due to the glucose-lowering effects of anti-diabetic medications (39% of T2D patients in our study) and other confounding factors.

There are different analytical methods available to measure ucOC in circulation, of which direct Glu-OC EIA kit and hydroxyapatite binding assay are most commonly used. Hydroxyapatite assay is based on the rationale that cOC binds to Ca^2+^ ions in hydroxyapatite with higher affinity than ucOC. Hydroxyapatite binding is, however, not completely selective and some binding of ucOC to hydroxyapatite takes place, which makes this only a semi-quantitative method [38]. On the other hand, Glu-OC EIA kit is reported to measure only totally uncarboxylated ucOC [14] and there is no information available about possible detection of partially carboxylated forms. Currently available data suggest that to act as a hormone, ucOC has to be uncarboxylated at site Glu17, but not at other sites [13, 14, 39]. Our assay will detect the active form of osteocalcin, uncarboxylated at Glu17. Different methodologies may measure differently uncarboxylated forms of osteocalcin, also fragments with variable degree of carboxylation [2], which may explain inconsistences between studies.

Human and rat osteocalcin sequences have only two amino acid difference on Fab-AP13 binding site V10-L25 [1] and our assay was indeed able to measure rat ucOC in addition to human ucOC. Rat osteoblasts were grown in the presence of warfarin and vitamin D. Vitamin D stimulated the expression of osteocalcin and post-translational gamma-carboxylation of osteocalcin was inhibited with warfarin, which blocks the vitamin K epoxide reductase enzyme [40] and leads to elevated ucOC production [39]. ucOC concentrations measured with our novel assay were increased in cells treated with warfarin, or warfarin and vitamin D, when compared to cells without warfarin treatment. This confirms that indeed, our assay measures biosynthetic ucOC produced in the osteoblasts.

The strengths of this study include carefully characterized antibodies, for which the epitope is known in detail and which can be easily produced in large quantities as recombinant protein in *E. coli*. The antibodies can be used to create immunoassays or used as tools for ucOC detection in research as well as in putative clinical applications. The detection of Fab-APs can be easily, e.g., performed by measuring AP activity or using FLAG tag in the recombinant protein. In this study, the detection of Fab-APs was performed with secondary anti-AP antibody, which was labeled for time-resolved fluorescence allowing highly sensitive ucOC detection. One of the limitations of the study is that all studied antibodies were targeted to an overlapping epitope which prevented the development of a two-site immunoassay using merely Fab-APs. Surprisingly, binder C-A12, isolated in the selection where the target antigen was altered between ucOC and cOC in different selections rounds, targeted almost the same region. By analyzing additional clones enriched in this selection, binders against other epitopes would likely to be obtained. To circumvent the problem of limited epitope diversity on assay development, we used a well-characterized monoclonal antibody 2H9 (with high affinity to both ucOC and cOC in biological samples) as a capture antibody along with our highly specific Fab-AP as a detection antibody. The lack of antibodies for other epitopes is probably due to the phage-display screening strategy, which targeted for the minor differences between cOC and ucOC located in specific region of the molecule. Another limitation is that we analyzed a cross-sectional set of samples and we can only evaluate associations and not causal relationships. Further, we did not have information about other parameters of glucose metabolism, except for fP-Glucose, since biobank datasets are incomplete. Other factors such as physical activity [41, 42] and metabolic syndrome [43] may further affect circulating osteocalcin. The samples were, however, carefully selected with specific criteria for inclusion (overnight fast) and exclusion (bone-active medication) and information about the other medications were available. We call for further validation in real-life cohorts with this promising immunoassay. Analytical validation according to the Clinical and Laboratory Standards Institute guidelines will be warranted when the assay will be applied to clinical use.

As a summary, in the present study, we developed specific antibodies and a reliable method to evaluate circulating levels of ucOC. We also present here that ucOC levels are negatively associated with fasting plasma glucose levels in adults, supporting the concept for interaction between bone and glucose metabolism, particularly osteocalcin carboxylation, in humans.

## Electronic supplementary material

Below is the link to the electronic supplementary material.

Supplementary file1 (DOCX 120 kb)
