# Supplementary material for: Recombinant Antibodies with Unique Specificities Allow for Sensitive and Specific Detection of Uncarboxylated Osteocalcin in Human Circulation
Source: Calcif Tissue Int. 2020 Aug 24;107(6):529–42. doi: 10.1007/s00223-020-00746-8 (PMC7593320; doi:10.1007/s00223-020-00746-8)
Supplement: Supplementary file 1 — Supplementary file1 (DOCX 120 kb) [file 223_2020_746_MOESM1_ESM.docx]

**Supplementary Material and Methods**

**Screening of ucOC-specific binders from scFv-AP library**

32 clones from ucOC library and 21 from tOC library were selected for verification, after which six clones from both libraries were selected for production in a soluble format. DNA from selected clones was isolated (Qiagen Plasmid Miniprep kit) and sequenced, all selected clones were unique. For soluble expression, scFv genes were cloned at SfiI sited to vector pLK06H [1] creating scFv fusion genes with bacterial alkaline phosphatase (AP). Ligated DNA was transformed in electrocompetent *Eschericia coli* XL1-Blue cells, and clones were inoculated in 5 ml of SB medium (0.05% glucose, 10 μg/ml tetracycline, 100 μg/ml ampicillin). For production of scFv-AP the cultures were induced at OD600 of 0.8 with 100 μM IPTG and grown overnight at 26°C with 300 rpm shaking. The culture samples were lysed by added lysozyme and freeze-thaw cycles as described by Stuknyte et.al 2013 [2]. The binding activity of soluble scFv-AP proteins was confirmed with an ELISA assay similar to the initial screening assay and best clone was selected.

**scFv-AP clone C-A12 detects tOC in human samples**

In a paraller experiment, scFv fragment with binding to ucOC and cOC was determined and a clone scFc-AP C-A12 was selected for further characterization. scFv-AP antibody C-A12 binding site was determined with the peptides described. Binding site was concluded to be N-terminal and within Trp5-Arg20 (Supplemental Fig. 1A). C-A12 did not bind to carboxylation site hence it recognizes both ucOC and cOC similarly. Because of the similar binding site with Fab-APs, antibodies could not be paired.

Assay performance was optimized: SA-plates were coated with bio-MAb-2H9 (200 ng/well). Next, 10 µl of standard or sample with 150 µl BSR containing 5mM EDTA was added for 120 min. C-A12 (50 ng/well) and Eu-anti-AP (100 ng/well) were added for 120 min. Finally, EFI was added and TR-FIA was measured. ucOC peptide in BSR was used as a standard.

Assay linearity and analyte recovery were analyzed by adding 20 ng/ml of ucOC peptide either in BSR or biological sample and performing serial dilutions. Assay was linear up to dilution 0.01 in BSR, 0.25 in both serum and plasma, which equals to 2 ng/ml (Supplemental Fig. 1B). As in Fab-APs, also in C-A12 immunoreactivity was lost with serum (30%) or plasma samples (20%) (Supplemental Fig. 1C). Total run variability (8.2%) was determined with 36 blood samples. Sample handling and immunoassay detectability was assessed as described. C-A12 based immunoassay was able to detect tOC in serum samples stored at RT up to 24 hours and +4℃ up to 6 hours (Supp Fig 1D) and at plasma samples the corresponding times were at RT 4 hours as well as at +4℃ 4 hours (Supplemental Fig. 1E). Interestingly, plasma samples were degraded by 15% in both samples stored at RT or +4℃ immediately (Supplemental Fig. 1E). There was no loss of immunoreactivity in either serum or plasma samples stored at -20℃ in the seven day time period observed. Novel immunoassay was compared with a validated tOC immunoassay based on MAbs 2H9/6F9 and using cOC (1-49) as a calibrator [3] using plasma samples (N=35). Concentrations measured with C-A12 based immunoassay were on average 0.34 fold lower than those measured with 2H9/6F9, due to differences in assay standardization, but concentrations observed with these two assays were significantly correlated to each other (Spearman’s correlation r=0.86, p<0.0001)

**Osteoblast culture *in vitro***

Animal experimentation was approved by the local review committee of Central Animal Laboratory, University of Turku (Turku, Finland). Female Sprague Dawley rats were sacrificed at age of 3-4 weeks and hind legs were extracted. Epiphysis of long bones were cut and bone marrow was flushed with needle. Bone marrow cells were cultured in αMEM medium (Gibco, USA), supplemented with 15% fetal bovine serum (Gibco), 10 mM HEPES (Gibco), 2mM GlutaMAX™ (Gibco), 100 u/ml Penicillin-Streptomycin (Gibco) and 10^-8^ M dexamethasone (Sigma). After 48 hours medium was changed and adherent, bone marrow stromal cells (MSC), were cultured for five days. MSCs were collected with 0.05% EDTA trypsin (Gibco) and seeded on either 24 well microwell plate with glass cover slip (4 000 cells/well) or on 6 well plate (80 000 cells/well) in osteogenic medium (αMEM medium with 10% fetal bovine serum, 2mM GlutaMAX™, 100µg/ml Penicillin-Streptomycin 10mM sodium β-glycerophosphate (Fluka BioChemika) and 70 µg/mL L-ascorbic acid 2-phosphate (Sigma)) for 9 days. On days 3 and 6 culture medium was changed and 25 µM 1,25(OH)_2_ dihydroxyvitamin D (Vitamin D, Sigma) and 25µM warfarin (Sigma) was added to medium. Medium samples were stored (-20℃) for further studies. On day 9, osteoblasts differentiated on glass were fixed with 4% PFA and stored at +5℃.

**Immunofluorescence staining of the osteoblasts**

Osteoblasts were permeabilized with 0.05% Triton x-100 in PBS and then blocked with 10% goat serum (ab7481, Abcam) in 0.05% Tween 20 in PBS for 60 min. 5µg/ml of Fab-AP13 or MAb-2H9 in 3% BSA in 0.05% Tween-20 was used as a primary antibody and incubated for 120 min. 5µg/ml anti-AP in 3%BSA in 0.05% Tween-20 was added to Fab-AP13 stained cells for 60 min. AlexaFluor488 labelled anti-rabbit for Fab-AP13 and anti-mouse for MAb-2H9 (1:1000, ab150077, ab150113, respectively, Abcam) in 3% BSA in 0.05% Tween-20 for 60 min. Samples were mounted with mounting solution Vectashield (Vector laboratories, Burlingame, California USA11). Cells were imaged with Zeiss Axioimager.

**References**

(1)Huovinen T, Syrjanpaa M, Sanmark H, Brockmann EC, Azhayev A, Wang Q, Vehniainen M, Lamminmaki U (2013) Two ScFv antibody libraries derived from identical VL-VH framework with different binding site designs display distinct binding profiles. Protein Eng Des Sel 26:683-693.

(2)Stuknyte M, Brockmann EC, Huovinen T, Guglielmetti S, Mora D, Taverniti V, Arioli S, De Noni I, Lamminmaki U (2014) Lactobacillus helveticus MIMLh5-specific antibodies for detection of S-layer protein in Grana Padano protected-designation-of-origin cheese. Appl Environ Microbiol 80:694-703.

(3)Kakonen SM, Hellman J, Karp M, Laaksonen P, Obrant KJ, Vaananen HK, Lovgren T, Pettersson K (2000) Development and evaluation of three immunofluorometric assays that measure different forms of osteocalcin in serum. Clin Chem 46:332-337.

**A**

**C**

**B**

**F**

**E**

**D**

**
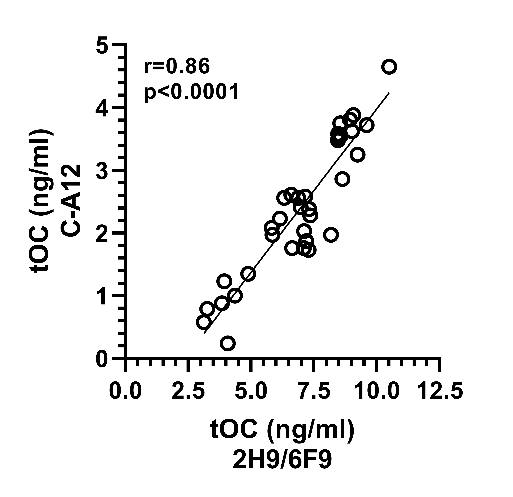
**

Supplemental Figure 1 Antibody binding site was determined for C-A12 (A). Linearity of dilution was determined by adding 20 ng/ml ucOC peptide in buffer (square), serum (cross) or plasma (plus) samples and performing serial dilutions (B) Also, regression of dilution was determined and immunoreactivity was lost when comparing serum and plasma samples to buffer (C). The effect of sample handling was assessed with serum (D) and plasma (E) samples at RT (black) +4℃ (white) and -20℃ (gray). C-A12 based immunoassay was compared with a validated tOC assay, 2H9/6F9, (F) and Spearman’s correlation is shown.
